# Genetic aspects of feed efficiency and reduction of environmental footprint in broilers: a review

**DOI:** 10.1007/s13353-017-0392-7

**Published:** 2017-03-24

**Authors:** Ewa Sell-Kubiak, Klaus Wimmers, Henry Reyer, Tomasz Szwaczkowski

**Affiliations:** 1 Departament of Genetics and Animal Breeding, Poznan University of Life Sciences, Wolynska st. 33, 60-637 Poznan, Poland; 2 Institute of Genome Biology, Leibniz Institute of Farm Animal Biology, Wilhelm-Stahl-Allee 2, 18196 Dummerstorf, Germany

**Keywords:** Chicken, Feed efficiency, Genomic selection, Greenhouse gases, Heritability

## Abstract

Currently, optimization of feed efficiency is one of the main challenges in improvement programs of livestock and poultry genetics. The objective of this review is to present the genetic aspects of feed efficiency related traits in meat-type chicken and possible ways to reduce the environmental impact of poultry meat production with effective breeding. Basic measures of feed efficiency are defined and the genetic background of these traits, including a review of heritabilities is described. Moreover, a number of genomic regions and candidate genes determining feed efficiency traits of broilers that were detected over the past decades are described. Classical and genomic selection strategies for feed efficiency in the context of its relationships with other performance traits are discussed as well. Finally, future strategies to improve feed digestibility are described as it is expected that they will decrease wastes and greenhouse gas emission. Further genetic improvement of feed efficiency, should be examined jointly with appropriate feeding strategies in broilers.

## Introduction

The increasing demand to produce meat rich in protein for the rising human population requires searching for efficient ways to breed livestock. Currently, the poultry meat sector is one of the most intensively growing agri-food sectors. According to the FAO/OECD prognosis for the year 2020, poultry meat will be the most produced in the world reaching nearly 140 million tons of meat. This indicates an increase of nearly 21% compared to 116 million tons of meat produced in 2016. In addition, costs of feed are not stable and fluctuate between years, which reinforces the importance of feed efficiency improvement. So far, after 30 years of selection toward improved growth rate and feed efficiency (FE), body weight (BW) of broilers at day 35 changed from 1.40 kg to 2.44 kg, whereas the feed consumption increased from 3.22 kg to 3.66 kg of feed in the same time duration (as reviewed by Siegel 2014).

Moreover, the environmental issues are a crucial concern in poultry industry, mainly due to problems with manure in regions with very dense production (Nahm 2007; Bolan et al. 2010; Mignon-Grasteau et al. 2010a), where emission from poultry production include: ammonia, phosphorus, nitrogen, carbon dioxide, and methane (FAO/OECD). Between 1988 and 2007, the decrease in greenhouse gas and other waste emissions by exploiting the genetic potential of broilers was shown to be on the level of 20% for methane, 23% for nitrous oxide, and 10% for ammonia (Hume et al. 2011). Nonetheless, the European Union regulations continuously demand the reduction of nitrate emissions on a farm and country level. Sustainability of poultry production is more important than ever with the globalization of agriculture competing for land, water, and other resources with different sectors as well as increasing knowledge of the environmental impact of agriculture. Thus, there is a need for breeding strategies that improve the feed efficiency and reduce the environmental impact of poultry meat production.

The aim of this review is to discuss the genetic aspects of feed efficiency in broilers and possible ways to reduce the environmental impact of poultry meat production with effective breeding.

## Measures of feed efficiency related traits

Feed efficiency (FE) depends on the relation between the feed intake (FI) — input — and the growth (or body weight gain) — output — of an animal, and was described by several traits (Table 1). While growth is a trait rather easy to obtain by weighing the animals at certain points in a lifetime, the feed intake is a more complex measure to obtain. The FI can be collected on an individual level by providing automatic feeding stations to the group-housed chickens (Howie et al. 2009; Howie et al. 2011) or by caging the birds individually. The group-housing allows measuring the FI on a large number of birds, for example, by applying the sound technologies (Aydin et al. 2014). The individual caging is cheaper in terms of data collection, however, it can create bias measures of FI as bird’s activity and feeding behavior are modified, because it lacks the social interaction between the birds, and thus it raises welfare issues (Shields and Greger 2013). It was shown that selection for high performance in broilers affects the feeding behavior of those animals as they exhibit a tendency to reach only for the free feed rather than search for the feed on the floor (Neves et al. 2010). Moreover, the level of FI is affected by the presence of other feeding birds (Collins and Sumpter 2007).

**Table 1:** Different measures/traits of feed efficiency

Measure/trait	Abbreviation	Formula^a^
Feed conversion ratio (gross efficiency)	FCR	FCR=FI/PO
Residual feed intake	RFI	RFI=FI – (α + β_1_ BW^0.75^ + β_2_ ΔBW)
Coefficient of digestibility	CDU	CDU = 100–100(DEW/FI)
Maintenance efficiency^b^	ME	ME=BW/FI_n_

^a^FI – feed intake, PO – production outputs, BW – body weight, BW^0.75^ – metabolic body weight, FI_n_ – feed intake at zero change of body weight, DEW – weighted dried excreta, α, β_1_, β_2_ – regression coefficients, ΔBW – change of body weight

^b^Maintenance efficiency is defined as the feed needed to maintain the energy requirements of the animal, i.e., to keep temperature level, body movement, and basal metabolism level, without affecting the body weight

### Feed conversion ratio

Feed conversion ratio (FCR) is one of the most widely used measures of FE. It is described as the ratio between feed inputs and product outputs. The FCR as a measure of FE was proposed in 1941 by Byerly and in the same year another study showed that it has a genetic variability (Hess et al. 1941), which indicated for the first time the potential of FCR to be improved by selection. It is recommended to consider FCR as age-constant to account for the growth rate differences at different ages (Chambers and Lin 1988).

### Residual feed intake

Residual feed intake (RFI) is defined as the difference between the measured feed intake and the expected feed intake of an animal and accounts for its maintenance requirement (on the basis of multiple regression equation). The expected feed intake is based on the production of the individual (see Table 1). The RFI was mentioned for the first time in the formula for feed consumption presented by Byerly (1941) in his study on laying hens, whereas Bordas and Mérat (1975) performed the first study and selection experiment on this trait in poultry.

The use of RFI over FI is preferred since it reflects the variation in efficiency of using feed by broilers (Kennedy et al. 1993). The RFI in more efficient animals has a negative value, which indicates lower energy requirements than predicted (Willems et al. 2013). It should to be noted that phenotypically it is independent from production traits and its mean in the population is zero (Kennedy et al. 1993).

### Coefficient of digestibility

The approach used for the calculation of FCR and RFI does not directly account for individual’s ability to digest specific nutrients. It is also possible to use the individually collected excreta and measure the level of digestibility of different feed components. However, this is a rather laborious task. Nonetheless, to be able to reduce the environmental footprint of the poultry sector, digestibility might be one of the most important traits. The coefficient of digestibility (CDU) represents the proportion of the particular nutrient (protein, lipid, starch, dry matter) that is actually digested and absorbed. The consideration of CDU provides the potential to focus on the reduction of poultry excretion of ammonia (De Verdal et al. 2013).

Recent studies on broiler lines selected for high and low apparent metabolized energy corrected for zero nitrogen retention (Mignon-Grasteau et al. 2004), showed nearly 50% lower excretion of nitrogen and nearly 40% decreased excretion of phosphorus in line with high apparent metabolized energy (Mignon-Grasteau et al. 2010b; De Verdal et al. 2011). A follow up study by De Verdal et al. (2013) focusing on genetic aspects of those results indicated that with selection for digestibility it is possible to reduce the environmental footprint of poultry breeding. Based on successful selection of the experiment performed on broilers on digestibility of a wheat-based diet (Mignon-Grasteau et al. 2004) and follow up studies, the CDU was applied to commercial breeding programs.

## Genetic background of feed efficiency traits

Through the introduction of FE measurements in poultry breeding, the studies on their genetic variation could begin. Table 2 presents an overview of studies reporting heritabilities of FE-related traits. In Table 3 the genetic correlations with production traits are shown. With the access to genomic tools and the possibility to genotype broilers, further studies were performed to detect genomic regions associated with FE-related traits. An overview of quantitative trait loci (QTL) and candidate genes is shown in Table 4. As mentioned, collecting feed intake data on a regular basis is not an easy task, and seven out of 16 described studies barely reached a 1000 observations (Table 2).

**Table 2:** Heritability estimates of most commonly used feed efficiency traits (feed conversion ratio, residual feed intake, and coefficient of digestibility)

Heritability estimate (with SE)^a^			Population	References
FCR	RFI	CDU		
0.20 (0.07) (sire population) 0.35 (0.09) (dam population)	–	–	Broiler sire (N=1832) and dam (N=1333) populations synthesized from nine broiler sire and seven broiler dam stocks – FCR not adjusted	Bernon and Chambers (1988)
0.16 (0.06) (sire population) 0.28 (0.09) (dam population)	–	–	Broiler sire (N=1832) and dam (N=1333) populations synthesized from nine broiler sire and seven broiler dam stocks – FCR adjusted for body weight	Bernon and Chambers (1988)
0.11 (0.20) 0.15 (0.14) 0.13 (adjusted for weight)	–	–	Two strains of broiler chickens (N=244 and N=1039) – FCR adjusted or not for body weight	Wang et al. (1991)
0.24 (not adjusted) 0.27 (adjusted for body weight at 28 days) 0.34 (adjusted for body weight at 28 and 42 days)			Three strains of broilers from nine generations of multitrait (2 strains derived from a “Cornish” broiler sire population N= 5077 and N=4238; 1 from a “White Rock” broiler dam population N=7428) – FCR as average of 3 strains	Chambers et al. (1994)
0.28 (0.04) (at 22°C) 0.27 (0.04) (at 32°C)	–	–	1521 male broilers reared from 4 to 6 weeks of age at 22° or 32°C	Beaumont et al. (1998)
0.07 (0.03)	–	–	901 Athens-Canadian randombred chickens from 28 to 35 days	Zhang et al. (2003)
0.29 (0.06)	0.49 (0.07)	–	2166 broilers from 23 to 48 days	Pakdel et al. (2005)
0.11 (0.07)	0.23 (0.10)	–	529 Campero-INTA broilers from 54 to 75 days	Melo et al. (2006)
0.33 (0.03)	0.45 (0.06)	–	1061 birds from a commercial slow-growing meat producing line	N’dri et al. (2006)
0.16 (0.03)	–	–	3189 male broiler line	Gaya et al. (2006)
0.41 (0.02)	0.42 (0.01)	–	2400 broilers from 35 to 42 days	Aggrey et al. (2010)
0.10 (0.03)	–	–	919 birds from 28 to 35 days	Ankra-Badu et al. (2010)
–	–	0.25–0.29 (wheat diet) 0.04–0.26 (corn diet)	820 F2 birds being cross of two broiler lines selected for high and low apparent metabolized energy corrected for 0 N retention	Mignon-Grasteau et al. (2010b)
–	–	0.30 (0.02) (wheat diet)	630 birds being cross of two broiler lines selected for high and low apparent metabolized energy corrected for 0 N retention	De Verdal et al. (2011)
0.30–0.43	–	–	14,000–18,000 birds from 4 lines	Howie et al. (2011)
0.10	0.35	–	2289 Arkansas random bred broiler control population	Aggrey et al. (2014)
–	0.14 (0.03)	–	2301 broiler chickens from 35 to 42 days	
–	0.41 (0.03)	–	450 Cross between fast growing male Arian line and Orumieh Iranian native fowl	Begli et al. (2016)

^a^FE – feed efficiency, FCR – feed conversion ratio, RFI – residual feed intake, CDU - coefficient of digestibility

**Table 3:** Genetic correlations with production traits of most commonly used feed efficiency traits: feed conversion ratio (FCR), residual feed intake (RFI), and coefficient of digestibility (CDU)

Trait	Genetic correlation with production traits	References
FCR	body weight at 38 days (0.07), body weight at 42 days (0.35), feed intake (0.38), leg/breast weight (0.10), liver weight (0.23), heart weight (0.16), abdominal fat content (0.35)	Gaya et al. (2006)
	leg yield (-0.70), abdominal fat yield (0.44), breast yield (0.00)	N’dri et al. (2006)
	metabolic body weight (0.57), body weight gain (-0.14), FI (0.54)	Aggrey et al. (2010)
	body weight (0.11)	Ankra-Badu et al. (2010)
	average daily feed intake (0.91)	Howie et al. (2011)
	body weight gain (-0.55), FI (0.45)	Aggrey et al. (2014)
RFI	body weight 4 wk (-0.18), body weight gain (-0.13)	Zhang et al. (2003)
	leg yield (-0.32), abdominal fat yield (0.44), breast yield (-0.35)	N’dri et al. (2006)
	metabolic body weight (0.45), body weight gain (0.06), feed intake (0.33)	Aggrey et al. (2010)
	body weight gain (-0.02), FI (0.29)	Aggrey et al. (2014)
	body weight 0–4 wk (0.17), body weight 5–6 wk (0.27)	González-Cerón et al. (2015)
CDU (total dry matter)	body weight at 23 days (0.16)	De Verdal et al. (2011)

**Table 4:** Quantitative trait loci (QTL) and single nucleotide polymorphisms (SNP) for feed efficiency traits (feed efficiency, feed conversion ratio, residual feed intake, and coefficient of digestibility) in chicken based on Chicken QTL database (http://www.animalgenome.org/QTLdb/chicken.html) and additional papers not included yet in this database

Chromosome	Trait^1^	QTL/SNP position (Mbs)	QTL span (cM)	Genetic variance explained by QTL (%)	Proportion of phenotypic variance explained by QTL	Candidate gene	Reference
**1**	FE FCR RFI	90.4–123.0 33.3–47.4 54.87 56.9–57.9 76.57 181.83 181.84 186.96 33.3–47.4	358–416 98–128 – 162.43–165.17 – – – – 98–128	– – 0.54 –	0.11 0.09 0.005 0.14	*–* *–* *HSP90B1* *AGK* *ZNF384* *NOX4* *ITA* *ITA* *–*	Hansen et al. 2005 ^b^ De Koning et al. 2004 ^c^ Shah et al. 2016 ^d^ Reyer et al. 2015 ^c^ Shah et al. 2016 ^d^ De Koning et al. 2004 ^c^
**3**	FCR RFI	13.99 39.02 108.2–109.5 51.8–64.9 102.8–102.9 103.8–108.2 108.2–109.5	– – 300–317 151.60–190.94 272.2 300–317 279–300	– – – – –	0.18 – – 0.10	*TMX4* *TSNAX* *–* *–* *–* *–* *–*	Shah et al. 2016 ^d^ De Koning et al. 2004 ^c^ Mignon-Grasteau et al. 2015b ^e^ Parsanejad et al. 2004 ^f^ De Koning et al. 2004 ^c^
**4**	FCR RFI	18.2–31.1 30.7–30.8 29.8–30.8 48.9–49.9 69.4 17.0–17.1 18.2–31.1 19.7 21.5–21.6	82–101 99.9 – 139.41–142.19 – 80 82–101 88.3 93.6	– – – 0.52 – – – –	0.09 0.04 – – 0.14 0.22	*–* *–* *HHIP* *PPP1R3B* *PGM2* *–* *–* *–* *–*	De Koning et al. 2004 ^c^ De Koning et al. 2003 ^c^ Reyer et al. 2015 ^c^ Shah et al. 2016 ^d^ De Koning et al. 2004 ^c^ De Koning et al. 2003 ^c^
**5**	FCR RFI	27.3–28.5 28.5–30.2 30.2–31.9	79–83 83–88 88–93	– – –	0.14 0.15	*–* *–* *–*	De Koning et al. 2004 ^c^
**6**	FCR	6.6–7.6 191.97	21.43–24.97-	0.95	0.01	*Sirt1* *ANKRD1*	Reyer et al. 2015 ^c^ Shah et al. 2016 ^d^
**7**	FCR RFI	6.67 6.81 22.6 25.6–25.6 25.7–26.6 26.6–26.6 26.6–28.7	– – – 33.72–120.48 120.56–125.25 92 92–109	– 0.63 0.53 – –	– 0.006 0.005 0.10	*COL18A1* *COL6A2* *PRKAG3* *EPB41L5* *EPB41L5* *–* *–*	Shah et al. 2016 ^d^ Jin et al. 2016 ^g^ Reyer et al. 2015 ^c^ De Koning et al. 2004 ^c^
**8**	FCR RFI	7.0–7.8 13.37 6.7–6.8	15–23 – 4	– –	0.14 0.11	*–* *TGFBR3* *–*	De Koning et al. 2004 ^c^ Shah et al. 2016 ^d^ De Koning et al. 2004 ^c^
**9**	FCR RFI	5.7–13.7 1.9–5.7 13.6–13.7	44–61 0–44 61	– – –	0.18 0.06	*–* *–* *–*	De Koning et al. 2004 ^c^
**10**	FCR RFI	3.16 19.4 19.1–20.3	– – 123.68–131.43	–	–	*IREB2* *CTDSPL2* *–*	Shah et al. 2016 ^d^ Mignon-Grasteau et al. 2015b ^e^
**11**	FCR	12.8–17.4	54–69	–	0.18	*–*	De Koning et al. 2004 ^c^
**12**	FCR RFI	8.97 16.4 16.4 16.4 16.5 16.7	– – – – – –	0.05 0.05 0.05 0.07 0.03	– – – – –	*IP6K2* *PDZRN3* *PDZRN3* *PDZRN3* *CHL1* *ZMPSTE24*	Shah et al. 2016 ^d^ Xu et al. 2016 ^h^
**13**	FCR RFI	12.91 15.3–18.3 16.3–16.4	– – 67	– –	– –	*HNRNPH1* *SLC22A4* *–*	Shah et al. 2016 ^d^ Mignon-Grasteau et al. 2015b ^e^ De Koning et al. 2004 ^c^
**14**	FCR	15.14				*–*	Shah et al. 2016 ^d^
**15**	FCR	9.7 9.7	– –	– –	– –	*PRKAB1* *PRKAB1*	Jin et al. 2016 ^f^
**16**	FCR CDU dry matter CDU starch CDU protein	– – – –	8 At telomere At telomere At telomere	– – – –	– – – –	*–* *–* *–* *–*	Ewald et al. 2007 ^i^ Tran et al. 2014 ^e^
**17**	FCR	0.17 7.5–8.5 7.5–8.5	– 55.41–63.20 55.41–63.20	0.74 –	– –	*–* *RXRA* *NR5A1, NR6A1*	Shah et al. 2016 ^d^ Reyer et al. 2015 ^c^
**19**	FCR RFI CDU dry matter	2.6–3.0 9.95 0.0–2.0 9–10	68.16–25.56 – 0.10–17.20 –	0.87 – 0.63	– – –	*GFT2I* *–* *CLDN3, CLDN4, EPCAM* *MED31, KIAA0753, SLC13A5, XAF1, TXNDC17*	Reyer et al. 2015 ^c^ Shah et al. 2016 ^d^ Mignon-Grasteau et al. 2015b ^e^ Van Goor et al. 2015 ^j^
**20**	FCR CDU dry matter CDU starch	0.45 – 12.9–13.9 –	– 9.0 – 9.0	– 0.62 –	– – –	*TGIF2* *–* *–* *–*	Shah et al. 2016 ^d^ Tran et al. 2014 ^e^ Van Goor et al. 2015 ^j^ Tran et al. 2014 ^5^
**21**	CDU dry matter	Not given 0.5–0.6	– –	0.59 0.53	0.12 –	*–* *–*	Bjorkquist et al. 2014 ^j^ Van Goor et al. 2015 ^j^
**22**	FCR	4.5	29.97–79.94	–	–	*ADRAIA*	Reyer et al. 2015 ^c^
**23**	RFI CDU starch	1.8 –	– 30.0	– –	– –	*–* *–*	Xu et al. 2016 ^h^ Tran et al. 2014 ^e^
**24**	FCR	0.2–2.8 0.2–2.8	1.81–28.91 1.81–28.91	– –	– –	*–* *–*	Mignon-Grasteau et al. 2015b ^e^
**25**	FCR	5.94	–			*MEF2D*	Shah et al. 2016
**26**	FCR CDU starch	0.1–2.5	0.80–36.79 36.0	– –	– –	*–* *–*	Mignon-Grasteau et al. 2015b ^e^ Tran et al. 2014 ^5^
**27**	FCR CDU dry matter	0.2–1.2 1.32 –	2.13–18.85 – 12.0	– –	– –	*–* *KIF18B* *–*	Mignon-Grasteau et al. 2015b ^e^ Shah et al. 2016 ^d^ Tran et al. 2014 ^e^
**Z**	FCR RFI	0.4–5.6 0.4–5.6 2.1–7.8	1.09–15.77 1.09–15.77 6.22–22.33	– – –	– – –	*–* *–* *–*	Mignon-Grasteau et al. 2015b ^e^

^a^FE – feed efficiency, FCR – feed conversion ratio, RFI – residual feed intake, CDU - coefficient of digestibility

^b^Cross of a Cornish meat-type strain 21 and an inbred egg-type strain WG

^c^Commercial broiler population

^d^Pure- Line chicks of Marshall Breed (indigenous broiler breed)

^e^Chickens from lines divergently selected on the basis of high or low digestive efficiency and crossed to produce an F2 design

^f^Non-inbred White Leghorn strain

^g^Two yellow meat-type chicken strains, N202 and N301

^h^Yellow-plumage dwarf chicken line N301

^i^Three commercial broiler lines

^j^F18 and F19 generations of broiler (heat-susceptible) x Fayoumi (heat-resistant) advanced intercross line

### Heritabilities

The heritability estimates of FCR varied in recent studies from 0.07 to 0.41 (Table 2). The variability of estimates is due to a number of reasons including breed of the birds, sex, age, diet, rearing environment, and the number of birds used in the study. Mentioned heritability levels indicate good potential for FCR to respond to selection, which is consistent with the success of selection improving this trait in the poultry breeding industry.

The heritability of RFI is reported on the level between 0.23 and 0.49 (Table 2). The RFI is often compared with FCR and some genetic studies indicate very high genetic correlation between those two traits from 0.74 to 0.93 (Van Bebber and Mercer 1994; Pakdel et al. 2005; Melo et al. 2006; N’dri et al. 2006; Aggrey et al. 2010).

Heritability estimates of CDU were reported in two studies revealing estimates in the range from 0.04 to 0.30 (Mignon-Grasteau et al. 2010; De Verdal et al. 2011). However, those heritabilities are estimated for animals fed different diets: wheat (Mignon-Grasteau et al. 2010; De Verdal et al. 2011) or corn (Mignon-Grasteau et al. 2010, see Table 2).

The heritability levels presented above illustrate that despite intensive selection to improve FE-related traits in the past 30 years, there is still potential to exploit the functional biodiversity contributing to the genetic variation in FE and providing possibilities to further improve this trait.

### Detected QTL and candidate genes

The QTLs for FE-related traits were so far reported on 24 out of 39 chicken chromosomes (GGA) including the sex chromosome Z (Table 4). The first studies were performed by de Koning in 2003 and 2004, which presented the first regions associated with FCR and RFI. Since then, a number of studies have indicated new genomic regions involved in all FE-related traits with the most recent for CDU. The QTLs as presented in Table 4 explained from 0.005 to 0.22 of a fraction of phenotypic variance of FE-related traits, with the highest values for RFI 0.06-0.22. However, very few QTLs overlap between the studies (Table 4). On GGA26, based on position in centiMorgans (cM), QTL for FCR detected by Mignon-Grasteau et al. (2015b) could be located at the same position as QTL for CDU of starch in Tran et al. (2014). Similar overlaps can be seen on GGA27 for QTL for FCR (Mignon-Grasteau et al. 2015b) and CDU of dry matter (Tran et al. 2014). It should to be noted that those two studies used animals coming from the same experimental population, which was selected for divergent digestive efficiency. Overlaps in position in detected QTLs can also be seen within a study (e.g., De Koning et al. 2004; Tran et al. 2014; Mignon-Grasteau et al. 2015b), which indicates that many detected QTLs for FE-related traits are breed and experiment specific.

The QTL studies presented in Table 4, often indicate possible candidate genes underlining the feed efficiency related traits. The candidate gene approach focuses on linking the known genes with the trait of interest. Reyer et al. (2015) performed a genome wide association study (GWAS) on 864 male broilers from a commercial line selected for feed efficiency and growth traits. In their study several candidate genes were found to be associated with FCR (Table 3); however, after additional analyses two of them were selected as the most promising ones. Those were *AGK* and *GTF2I* representing different biological processes affecting FCR in broiler. Specifically, the *AGK* gene encodes a mitochondrial acylglycerol kinase, catalyzing the synthesis of phosphatidic and lyso-phosphatidic acids, which are used as signalling molecules. Moreover, the observed effects of the *AGK* on FCR were suggested to be linked with mitochondrial function which is in agreement with previous observations linking feed efficiency and mitochondrial activity (Bottje et al. 2002). They also noticed the effect of different alleles of *AGK* gene on variation of body weight and weight gain in animals. The *GTF2I* gene is shown to be associated with growth traits and overall effect on weight in different species and is suggested to be involved in the transcriptional regulation of growth factor signaling pathway (Hakre et al. 2006).

In a large selection experiment, where the 8^th^ generation of high and low digestive efficiency lines were crossed to produce 820 F2 birds, Mignon-Grasteau et al. (2015b) detected several QTL for FE-related traits. They found one candidate gene for FE and three for RFI. The gene associated with FE was *SLC22A4* (*solute carrier family 22 member 4*). The encoded protein is involved in the transport of ergothionein, which is suggested as a protective molecule of the intestine. Accordingly, *SLC22A4* knock-out mice showed degradation of structures of the small intestine (Kato et al. 2010). The genes associated with RFI were *CLDN3*, *CLDN4*, and *EPCAM* and are involved in the claudin pathway, which is a vital member of the tight junctions between intestinal cells (Mattel et al. 2005; Lei et al. 2012).

Beside the genome-wide analyses, targeted approaches revealed several genetic variants of functional candidate genes for FE-related traits in different meat-type chicken populations (Nie et al. 2005). These genes contribute to a complex network of genetic factors influencing diverse biological processes with impact on FE-related traits. Moreover, recent analyses also provided evidence for the impact of sequence variants in non-coding RNAs on FE as shown by a SNP in the microRNA miR-1596 locus which was significantly associated with residual feed intake (Luo et al. 2015).

Studies mentioned above and in Table 4 represent only a small proportion of genetic factors with contribution to the genetic architecture of FE-related traits and influence only distinct biological pathways involved in those traits (Herd and Arthur 2009). The high number of presented candidate genes and localization of QTLs suggest that those traits are highly polygenic, and genes controlling FE-related traits are involved in various biological processes. Thus, Table 5 shows the ontology of possible candidate genes performed with a web tool of Princeton University (http://go.princeton.edu/). Grouping by processes in which the genes are involved indicated that *COL18A1, EPB41L5, CLDN3* regulate the epithelial cell morphogenesis, *Sirt1, NR5A1, RXRA*, and *NR6A1* mediate the signaling pathway and cellular response to steroid hormones, whereas *Sirt1, HHIP, TGFBR3, NR5A1, EPB41L5, RXRA*, and *NR6A1* regulate the cellular response to endogenous stimuli. When it comes to the biological function of the candidate genes only *NR5A1, RXRA*, and *NR6A1* were clearly indicated, but for three different functions: RNA polymerase II transcription factor activity, ligand-activated sequence-specific DNA binding; transcription factor activity, direct ligand regulated sequence-specific DNA binding; steroid hormone receptor activity. As presented in Table 5, candidate genes are enriched in cellular components of the apicolateral plasma membrane, RNA polymerase II transcription factor complex or in organelle. It should to be noted that only a small number of ale presented candidate genes indicated overlap in their ontology evaluation (Table 5).

**Table 5:** Ontology of the candidate genes grouped by process, function, and component performed with a web tool of Princeton University (http://go.princeton.edu/)

Gene ontology term	Corrected P-value	Genes annotated to the term
By process		
epithelial cell morphogenesis	0.001	COL18A1, EPB41L5, CLDN3
cellular response to endogenous stimulus	0.006	Sirt1, HHIP, TGFBR3, NR5A1, EPB41L5, RXRA, NR6A1
steroid hormone mediated signaling pathway	0.006	Sirt1, NR5A1, RXRA, NR6A1
cellular response to steroid hormone stimulus	0.01	Sirt1, NR5A1, RXRA, NR6A1
single-organism process	8e-06	NR5A1, MED31, PRKAB1, PRKAG3, XAF1, KIF18B, CLDN4, IP6K2, HHIP, EPB41L5, TGIF2, PPP1R3B, ITA,ZMPSTE24, TGFBR3, CLDN3, SLC13A5, AGK, TMX4, CHL1, TXNDC17, Sirt1, COL18A1, PDZRN3, RXRA, PGM2,NR6A1
single-organism cellular process	0.006	NR5A1, PRKAG3, XAF1, KIF18B, IP6K2, HHIP, EPB41L5, TGIF2, PPP1R3B, ITA, ZMPSTE24, TGFBR3, CLDN3, AGK,TMX4, CHL1, Sirt1, COL18A1, PDZRN3, RXRA
By function		
RNA polymerase II transcription factor activity, ligand-activated sequence-specific DNA binding	0.001	NR5A1, RXRA, NR6A1
transcription factor activity, direct ligand regulated sequence-specific DNA binding	0.001	NR5A1, RXRA, NR6A1
steroid hormone receptor activity	0.003	NR5A1, RXRA, NR6A1
By component		
apicolateral plasma membrane	0.003	CLDN4, CLDN3
RNA polymerase II transcription factor complex	0.007	NR5A1, MED31, RXRA
membrane-bounded organelle	0.002	NR5A1, MED31, PRKAB1, IP6K2, KIF18B, EPB41L5, TGIF2, ITA, ZMPSTE24, TGFBR3, CLDN3, AGK, TMX4,CHL1, TXNDC17, TSNAX, Sirt1, COL18A1, PGM2, RXRA, NR6A1
organelle	0.00911	NR5A1, MED31, PRKAB1, IP6K2, KIF18B, EPB41L5, TGIF2, ITA, ZMPSTE24, TGFBR3, CLDN3, AGK, TMX4,CHL1, TXNDC17, TSNAX, Sirt1, COL18A1, PGM2, RXRA, NR6A1

## Selection strategies for feed efficiency affecting other production traits

### Classical approach

Arthur and Albers (2003) greatly summarized the most crucial gains in the knowledge and technologies for poultry breeding. Starting with the mass selection in 1900, hybridization and recording of the pedigrees in 1940, through artificial insemination in 1960, selection index in 1980, the shift from family feed conversion testing from 1970 to individual FCR testing in 1980, and finally creation of the Best Linear Unbiased Prediction (BLUP) in 1990 to estimate the breeding values. All of those methods had a huge effect on the success of selection to improve FE-related traits in poultry. In broilers the high number of offspring information that can be used as a source of information in classical approaches of selection strategies, can nearly double the genetic improvement over a generation compared to the same strategies applied to cattle (Jonas and de Koning 2015).

The FCR has been directly included into commercial breeding programs for decades and on average a change of 1.2% per generation was reported (Wall et al. 2010). It was presented both in experimental and commercial setting that FCR can be influenced by selection leading to an improvement in this trait from on average 2.30 kg/kg in 1985 to 1.50 kg/kg in 2010 (Siegel 2014). This increase in efficiency was possible mainly because of genetic improvement of the growth rate and the feed intake with the benefits of high energy diets (Brameld and Parr 2016). The FCR is a ratio of two traits and as such the direct selection on this trait should be avoided. It was shown, that during the direct selection for traits being ratios, the main focus is on the information from the numerator (here feed intake) despite other components (Gunsett 1984). This could lead to unbalance selection for FI and weight gain (Willems et al. 2013). In case of FCR, a faster genetic improvement can be achieved when selecting for FI and weight gain separately (Campo and Rodriguez 1990). Furthermore, FCR and weight gain are associated with ascites, sudden death syndrome, reduced immune competence, tibial dyschondroplasia, reduced reproductive performance, and other metabolic disturbances, which are important welfare and economic aspects of poultry breeding (Emmerson 1997).

The RFI is a trait that could be more easily incorporated into multi-trait selection indexes of commercial breeding companies as it is independent from other production traits (Willems et al. 2013). However, when selecting for the improved RFI a certain tradeoff needs to be kept in mind; since, the improvement in RFI means an increase in productivity leading to a decrease in FI based on genetic correlations between the two traits and due to a preferred selection of fast growing animals in meat production systems (Willems et al. 2013). The suggested alternative to maintain a desired level of RFI would be the selection of slower growing animal consuming less feed (Berry and Crowley 2012).

### Genetic relationships between FE-related traits and other production traits

Although the heritabilities of FCR and RFI indicate good potential for selection response and the consideration of CDU could further reduce the environmental impact of poultry breeding, the relation between FE-related traits and other production traits needs to be kept in mind while selecting for improved FE (Table 3).

The genetic correlations between FCR and body weight (BW) related traits varies depending on the study. Partially, it is related to the fact that different BW traits are used across those studies. The FCR is moderately correlated with body weight and negatively with BW gain (Table 3). The FCR with feed intake has correlations varying from 0.38 to 0.54 (Gaya et al. 2006; Aggrey et al. 2010; Aggrey et al. 2014) and with abdominal fat content from 0.35 to 0.44 (Gaya et al. 2006; N’dri et al. 2006). The moderate correlations indicate that the traits can be improved simultaneously, but some correlated response will be present when selecting for those traits separately. The RFI is positively genetically correlated with metabolic BW (0.45, Aggrey et al. 2010), abdominal fat content (0.44, N’dri et al. 2006), and feed intake as shown in two different studies: 0.33 (Aggrey et al. 2010) and 0.29 (Aggrey et al. 2014). The genetic correlation between RFI and BW gain is close to zero: 0.06 (Aggrey et al. 2010) and -0.02 (Aggrey et al. 2014).

So far little is known about relationships between CDU and production traits. One study reported the genetic correlation between CDU and BW at the 23^rd^ day of age on the level of 0.16 (De Verdal et al. 2011). Moreover, whereas the genetic correlation between FCR and RFI was shown to be very high and positive (Table 2), the genetic correlation between CDU for dry matter and FCR was reported on the level of -0.98 (De Verdal et al. 2011). This high negative correlation is very desirable as it is beneficial that FCR will decrease, whereas the CDU will increase. It also shows, that the two traits could be improved simultaneously by selecting only on one of them.

### Genomic selection strategies for feed efficiency

The biggest shift in poultry breeding were the DNA based technologies (Hocking 2010). The most important step toward learning about the genetic background of the biology and evolution of the chicken was the first release of the chicken genome sequence (Hillier et al. 2004). Since then, a number of different SNP-chips were developed to serve as a new technology in chicken breeding programs. The QTL studies presented in the previous section used different strategies (microsatellites, genome wide association, QTL study) and detected a number of QTLs for FE-related traits; however, they all had rather small effects on feed genetic variability of efficiency traits (Table 4, Chicken QTL database, http://www.animalgenome.org/QTLdb/chicken.html). In breeding practice, application of many genomic regions with small effects into a selection schemes might be difficult or even not feasible. Another way to utilize genomic information is the genomic selection, which is considered one of the essential methodologies in achieving the demands of a growing poultry meat sector (Fulton 2012). Even though the FE was already greatly improved with the classical selection methods, the genomic selection provides possibilities for further improvement of that trait. Moreover, the decrease in generation interval from 12 to 6 months can be expected with application of genomic selection as breeders do not need to obtain phenotypes for all young birds (Jonas and de Koning 2015).

In genomic selection, many SNPs are used at the same time to improve the estimated breeding values of selection candidates. Such genomic evaluation is also used to describe the relationship of the animals by constructing relationship matrices: **G**-matrix for all genotyped animals (Van Raden 2008; Yang et al. 2011) or **H**-matrix for both genotyped and ungenotyped animals in the population (Forni et al. 2011). The difference in genomic evaluation with **G** or **H** is that the **H** combines both pedigree and genomic relationship matrix and reveals pedigree- and genomic-based breeding values (GEBV). The **G**-matrix is based exclusively on the marker information and gives direct genomic breeding values (DGV). In brief the genomic selection can be described with those four steps:

1. Establish reference population – animals with observations/phenotypes and SNP genotypes.
2. Develop prediction equation (genotype <-> phenotype relationship).
3. Validate estimated breeding values in the reference population.
4. Apply on selection candidates – predict breeding value based only on SNP genotypes.

The genomic evaluation of an animal gives a more precise indication of the genetics underlying the observed phenotype, leading to an increased accuracy of the estimated breeding values (EBV). Moreover, the genomic selection provides estimates of breeding values to non-phenotyped selection candidates. This is important for production traits such as feed efficiency, since they are recorded later in life. In addition, the methodologies based on genomic information help to control the inbreeding level and remaining genetic variation of the traits. So far only one study applied genomic prediction to FE-related trait, which was performed with Bayesian approach on 394 birds genotyped for 4k SNP for which the phenotypic data on FCR were recorded (González-Recio et al. 2009). González-Recio et al. (2009) reported astonishing improvement of four times the accuracy of selection compared with the classical pedigree information.

Despite its clear benefits the genomic selection also brings some obstacles. One of them is still the costs of genotyping. Currently the cost of genotyping one animal is roughly $200, compared to the value of the broiler itself the costs are still very high (Fulton 2012). Moreover, to start implementing this technology into the breeding program it is necessary to genotype at least 3000-5000 animals in the training set. Thus, to achieve expected improvement in FE in broilers, the genotyping strategies have to be selected carefully to obtain visible improvement in efficiency with the maximal use of available genomic information (Avendaño et al. 2010).

## Reducing the environmental footprint

As mentioned earlier, the selection to improve feed efficiency in broilers decreased the emission of greenhouse gases and other wastes in poultry meat production (Hume et al. 2011). However, the poultry industry still remains the second largest (after cattle) producer of ammonia, phosphorus, nitrogen, carbon dioxide, and methane (FAO/OECD), and with the growing population of broilers the reduction of wastes is still an important issue. To further achieve an appropriate rate of breeding progress in FE-related traits, which would result in much decreased waste levels and feed needed for growth, it is necessary to look not only at the overall feed efficiency, but also on genes that affect the utilization of different nutrients (Reyer et al. 2015). It was shown that selection of broilers to digest wheat can decrease the dry and fresh excreta by -61% and -56%, respectively, and excretion of nitrates and phosphates by -13% and -30%, respectively, compared to birds selected for low digestive efficiency (De Verdal et al. 2013). Zhang et al. (2003) also conducted a study on phytate phosphorus utilization and their reduction in diet of Athens-Canadian random bred chickens, however, no clear recommendations for breeding programs were made. The answer to further improvements of waste reduction in broiler breeding might be based on strategies to exploit the composition of the microbiota.

Microbiota can be defined as the complex community of microbes that coexist internally and externally with an animal (Oakley et al. 2014; Qaisrani et al. 2015), and can be seen as a link between their diet and health (Sergeant et al. 2014) as it helps in assimilating nutrients, producing vitamins and amino acids (Apajalahti 2005). Furthermore, the undigested protein in a birds digestive system can become a source of amino acids for microbiota living in the gut (Qaisrani et al. 2015) as well as a bird itself (Obst 1989). Studies have indicated that microbiota is involved in nitrogen recycling by its ability to breakdown uric acid (Karasawa 1999), whereas ammonia utilization is possible by its transformation into bacterial protein (Rist et al. 2011). Both abilities of microbiota are important aspects of nitrate utilization in poultry breeding (Qaisrani et al. 2015). Currently, more than 640 different bacterial species in chicken gut are known (Apajalahti 2005). Modern technologies based on genomic information allow separating the microbiota from the host and studing its effect on the immune system and its importance for poultry nutrition including FE-related traits (Oakley et al. 2014; Mignon-Grasteau et al. 2015a). It is expected that understanding the host-microbial interactions between microbiota and chicken could improve feed digestibility, and decrease wastes and greenhouse gases emission (Sergeant et al. 2014). Therefore, further genetic improvement of feed efficiency to reduce the environmental footprint should be examined jointly with microbiome studies.
